# Clinicopathological significance of claudin-4 in gastric carcinoma

**DOI:** 10.1186/1477-7819-11-150

**Published:** 2013-07-04

**Authors:** Jin-Liang Zhu, Peng Gao, Zhen-Ning Wang, Yong-Xi Song, Ai-Lin Li, Ying-Ying Xu, Mei-Xian Wang, Hui-Mian Xu

**Affiliations:** 1 Department of Surgical Oncology and General Surgery, First Hospital of China Medical University, 155 North Nanjing Street, Heping District, Shenyang City 110001, China; 2 Department of Tumor Pathology and Surgical Oncology, First Hospital of China Medical University, Shenyang, China

**Keywords:** Gastric carcinoma, Claudin-4, Tight junctions

## Abstract

**Background:**

Aberrant expression of claudin proteins has been reported in a variety of cancers. Previous studies have demonstrated that overexpression of claudin may promote tumorigenesis and metastasis through increased invasion and survival of tumor cells. However, the prognostic significance of claudin-4 in gastric cancer remains unclear.

**Methods:**

Immunohistochemistry was used to analyze the expression of claudin-4 in 329 clinical gastric cancer specimens and 44 normal stomach samples, 21 intestinal metaplasia samples, and 21 adjacent precursor lesions dysplasia samples. Statistical analysis methods were used to evaluate the relationship between claudin-4 expression and various clinicopathological parameters. Univariate and multivariate analyses were performed, respectively, to detect the independent predictors of survival.

**Results:**

Claudin-4 expression was present in only 7(15.9%) normal gastric samples, but expression of claudin-4 in the intestinal metaplasia lesions and dysplasia lesions was 90.5% and 95.2%, respectively. The expression of claudin-4 was significantly associated with histological differentiation (*P *< 0.001) and tumor growth patterns (*P *< 0.001) but not associated with patient survival. However, intermediate type staining of claudin-4 exhibited a trend of correlation with patients’ survival (*P *= 0.023). The five-year survival rate with low expression of claudin-4 in intermediate type (76.4%) was similar to expanding type (64.5%), while the high expression group (46.6%) was closer to infiltrative type (50.7%).

**Conclusions:**

The findings in this study demonstrate claudin-4 aberrant expression in gastric cancer and precursor lesions. The expression of claudin-4 could serve as a basis for identifying gastric cancer of the intermediate type.

## Background

Gastric cancer is one of the most common malignancies, and approximately 738,000 deaths occurred due to gastric cancer in 2008 worldwide. Over 70% of new cases and deaths occur in developing countries, particularly in Eastern Asia [1]. Gastric cancer is generally understood to develop as a multistep progression from chronic gastritis, atrophic gastritis, intestinal metaplasia, dysplasia, and finally cancer [2]. During this process, loss of cell polarity and disruption of cell-cell junctions is frequently observed, and this plays an important role in cancer progression.

Tight junctions (TJs) are critical for maintaining normal structure and physiological function of the epithelium and endothelium [3,4]. TJs not only serve as a physical barrier to prevent solutes and water from passing freely through the paracellular space between epithelial and endothelial cell sheets, but also play critical roles in maintaining cell polarity and signal transduction [3,5,6]. However, cancer cells frequently exhibit abnormal TJ function [7].

Claudins are members of a large family of transmembrane proteins that are among the essential components of TJs, and claudin-aberrant expression potentially leads to structural and functional damage of TJs [8]. Presently, a total of 24 claudin genes have been identified in mammals, and they often show tissue-specific patterns of expression [9]. Recently, numerous studies have demonstrated the aberrant expression of claudins in several human cancers [10,11]. To our knowledge, the expression of Claudin-1, 2, 3, 4, 5, 6, 7, 18 and 23 have been reported in gastric cancers [12-23]. Among the various claudin proteins related to gastric cancer, the function of claudin-4 was not consistent. For example, Jung *et al*. [15] and Ohtani *et al*. [19] found that the expression of claudin-4 significantly correlated with favorable survival for patients with gastric cancer in 72 and 124 cases, respectively. However, Resnick *et al*. [13] reported that moderate to strong claudin-4 staining in gastric cancer was significantly associated with poor survival in 146 cases. Soini*et al*. [14] found no clear association between claudin-4 expression and patient prognosis in 118 cases. Interestingly also, the expression of claudin-4 has been shown to play a role in determining matrix metalloproteinase(MMP) activity, indicating that claudin-4 might have mediated invasion through the activation of MMPs [24,25]. Furthermore, previous studies reported that claudin-4 may have potential as a treatment for cancer [26-28]. Thus, further investigations are required to clarify these controversial results and the real functions of claudin-4. In this study, we aimed to identify the clinicopathological associations and prognostic value of claudin-4 expression in gastric cancer.

## Methods

### Patients and tissue samples

In this study, tissue specimens from a total of 329 patients with gastric cancer were obtained between 1998 and 2004 at the Department of Surgical Oncology, The First Hospital of China Medical University. All patients underwent curative radical gastrectomy with standard lymph node dissection. The histopathological diagnosis of 21 intestinal metaplasia, 21 dysplasia, 44 microscopically normal stomach mucosa, and 329 gastric adenocarcinoma samples from the 329 patients was performed by two independent pathologists. We obtained written informed consent from all patients, and the study was approved by the ethics committee of the China Medical University. None of the patients had received chemotherapy or radiotherapy before the surgical procedure. The detailed postoperative pathology diagnosis reports were obtained and included gender, age, tumor location, size, differentiation status, growth pattern, and tumor-node-metastasis (TNM) stage. The criteria of TNM classification for gastric carcinoma were in accordance with the 7th 2010 American Joint Committee on Cancer (AJCC) staging manual [29]. The criterion for histopathological grading of differentiation was in accordance with the 2000 world health organization classification of gastric carcinoma [30]. The criterion for the pattern of tumor growth was in accordance with the Japanese classification of gastric carcinoma, 3rd English edition. All patients’ characteristics are summarized in Table 1. The patient sample comprised 238 male and 91 female patients with a mean age of 57 years (range 26 to 81 years). All patients were followed up via telephone inquiry or questionnaire, and the followup time ranged from 1 to 136 months (median of 56 months).

**Table 1:** Clinicopathologic characteristics of 329 patients with gastric carcinoma

**Variable**	**Value**
Age at surgery,y	
Mean	57.0
Range	26-81
Gender, number	
Male	238
Female	91
Tumor size, cm	
Mean	5.0
Range	1.0, 15.0
Tumor location, number	
Upper	31
Middle	53
Lower	245
Differentiation status, number	
Well-differentiated	47
Moderately differentiated	54
Poorly differentiated	218
Undifferentiated	10
Growth pattern, number	
Expanding	76
Intermediate	81
Infiltrative	172
Tumor (T) stage, number	
T1	44
T2	52
T3	164
T4	69
Node (N) stage, number	
N0	100
N1	39
N2	76
N3	114
Lymphatic invasion, number	
Negative	248
Positive	81
Tumor stage, number	
IA, IB	63
IIA, IIB	86
IIIA, IIIB, IIIC	180
Vital statistics, number	
Alive	152
Dead, all causes	177
Dead, gastric cancer	143
Dead, unrelated	22
Information unavailable	12

### Immunohistochemistry

The formalin-fixed and paraffin-embedded (FFPE) tissue blocks were sliced into 5-μm-thick sections, then deparaffinized with xylene and rehydrated using a graduated series of ethanol. The sections were incubated in boiling citric acid buffer (pH 6.0) for antigen retrieval in a steam pressure cooker. The sections were incubated overnight at 4°C with the following primary antibodies: anti-claudin-4 monoclonal antibody, 1:100 dilution, clone 3E2C1, Zymed Laboratories Inc., CA, USA. Immunohistochemical staining was conducted using the MaxVision^TM^HRP-Polymer anti-Mouse/Rabbit IHC Kit (Fuzhou Maixin, China) with 3 amino-9-ethyl carbazole (AEC) as the enzyme substrate. The sections were then lightly counterstained with hematoxylin. Positive controls were colonic mucosa and the specimens in which the primary antibody was replaced with non-reactive antibodies served as negative controls.

Immunostaining results were interpreted independently by two pathologists using a semi-quantitative scoring system [31]. The immunostaining reactions were evaluated by staining intensity (0, no stain; 1, weak; 2, moderate; 3, strong) and the percentage of stained epithelial cells (0, < 5%; 1, 5 to 25%; 2, 26 to 50%; 3, 51 to 75%; and 4, >75%). The percent positivity of epithelial cells and staining intensity were then multiplied to generate the immunoreactivity score (IS) for each case. Specimens were rescored if there were discrepancies in IS between the two pathologists, until a consensus was reached. We divided the samples into two groups based on the results of the immunostaining in the tissues: low expression (IS < 4) and high expression (IS ≥ 4). This evaluation system for claudin-4 has been used in previous studies [32].

### Statistical analysis

A meta-analysis was performed to confirm the role of claudin-4 in gastric cancer. We searched the English literature for relevant studies published before June 1st, 2013, using the PubMed database with the following terms: ‘stomach neoplasm’ and ‘claudin-4’ in medical subject headings (MeSH). References in the retrieved articles were further screened for earlier original studies. The inclusion criteria were patients with gastric cancer, including a prognostic comparison between high and low expression of claudin-4. The corresponding authors were contacted to obtain missing information, and some studies were excluded if critical information was still missing after repeated requests.

For the quantitative aggregation of survival results, the observed-expected (O-E) statistic and variance were combined to give the effective value. The O-E values and the variances were estimated from available data using the methods reported by Tierney *et al*. [33]. If the study provided a hazard ratio (HR), the O-E values and variances were estimated based on that. If the study did not provide a HR but reported the data in the form of a survival curve, survival rates were extracted at certain specified times to reconstruct an estimated HR and its variance.

We used the chi-squared test to evaluate the relationship between claudin-4 expression and various clinicopathological parameters. Survival analysis was performed using the Kaplan-Meier method, and differences between the groups were analyzed using the log-rank test. The Cox regression multivariate model was used in a stepwise forward manner to detect the independent predictors of survival. Two-tailed *P*-values less than 0.05 were considered to indicate a statistically significant result. All statistical analyses were performed using SPSS software (version 17.0; SPSS for Windows, Chicago, IL, USA) and RevMan 5.2 analysis software (The Cochrane Collaboration).

## Results

**Figure 1:**
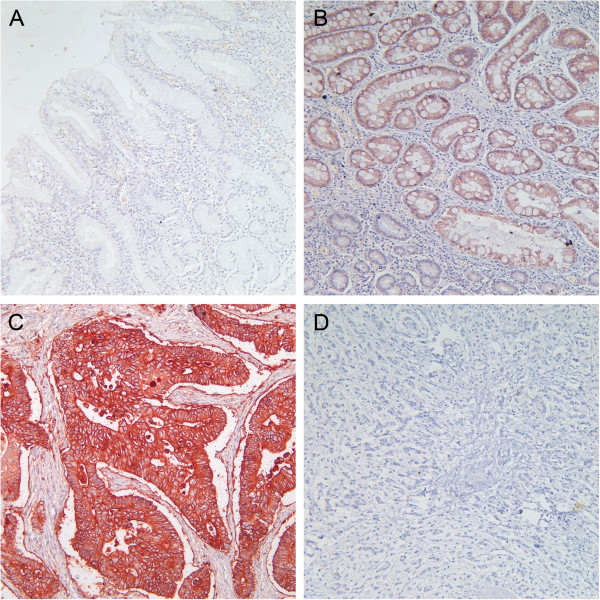
**Claudin-4 immunostaining. (A)** Normal gastric muscoma, no staining; **(B)** intestinal metaplasia, high expression; **(C)** moderately differentiated to well-differentiated gastric cancer, high expression; **(D)** poorly differentiated gastric cancer, no staining. Magnification × 200.

Claudin-4 immunostaining occurred in a predominantly membranous pattern, with some samples displaying a low level of cytoplasmic staining. Only 15.9% (7/44) of normal gastric mucosa exhibited high expression levels of claudin-4. In contrast, 90.5% (19/21) of the intestinal metaplasia lesions and 95.2% (20/21) of the gastric epithelial dysplasia lesions exhibited high expression levels of claudin-4. Of the gastric carcinoma samples investigated, 53.2% (175/329) of cases demonstrated high claudin-4 expression (Figure 1).

**Figure 2:**
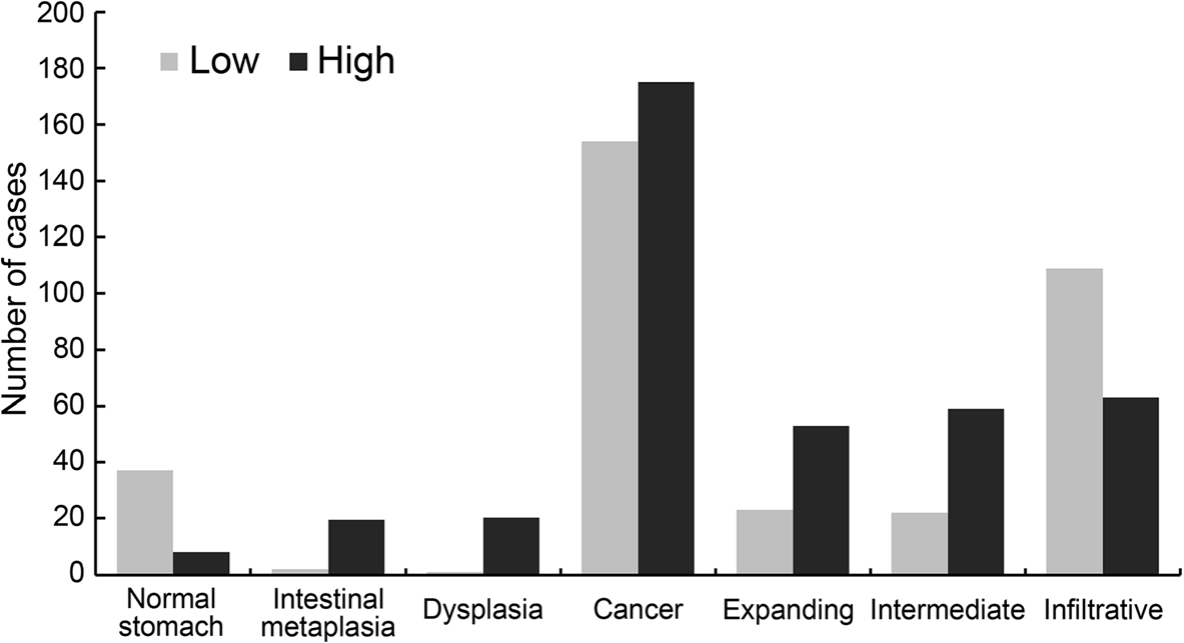
Expression of claudin-4 in normal stomach mucosa, intestinal metaplasia, dysplasia and cancer (including expanding, intermediate and infiltrative types).

The expression level of claudin-4 was significantly correlated with tumor differentiation (*P *< 0.001), gender (*P *= 0.003), age (*P* = 0.025) and tumor location (*P* = 0.033). According to microscopic inspection of the tumor growth pattern, 76 cases were classified as the expanding type and 172 cases as the infiltrative type, whereas 81 cases were determined to be the intermediate type. The expression level of claudin-4 was also significantly correlated with the tumor growth pattern (*P *< 0.001). High expression of claudin-4 was observed in 69.7% of the expanding type and 72.8% of the intermediate type of gastric cancer, whereas only 36.6% of the infiltrative type exhibited high expression. However, our findings revealed no significant correlation between the expression of claudin-4 and tumor size, depth of invasion, lymph node metastasis, lymphatic invasion, and tumor stage (Figure 2, Table 2).

**Table 2:** Statistical results of relationships between claudin-4 expression and various clinicopathologic characteristics

**Variable**	**Total patients(%)**	**Patients with low claudin-4 expression (%)**	**Patients with high claudin-4expression (%)**	***P-*value**
	329	154(46.8)	175(53.2)	
Age at surgery,y				0.025
≤ 60	192	100(52.1)	92(47.9)	
> 60	137	54(39.4)	83(60.6)	
Gender				0.003
Male	238	99(41.6)	139(58.4)	
Female	91	55(60.4)	36(39.6)	
Pathophysiologic features				
Tumor size (cm)				0.055
≤ 5	199	102(51.3)	97(48.7)	
>5	130	52(40.0)	78(60.0)	
Tumor location				0.033
Upper	31	9(29.0)	22(71.0)	
Middle	53	31(58.5)	22(41.5)	
Lower	245	114(46.5)	131(53.5)	
Histological type				<0.001
Differentiated (WD,MD)	101	24(23.8)	77(76.2)	
Undifferentiated (PD,UD)	228	130(57.0)	98(43.0)	
Growth pattern				<0.001
Expanding	76	23(30.3)	53(69.7)	
Intermediate	81	22(27.2)	59(72.8)	
Infiltrative	172	109(63.4)	63(36.6)	
T stage				0.147
T1/2	96	51(53.1)	45(46.9)	
T3/4	233	103(44.2)	130(55.8)	
Lymph node metastasis				0.905
Negative	101	48(47.5)	53(52.5)	
Positive	228	106(46.5)	122(53.5)	
Lymphatic invasion				0.898
Negative	248	117(47.2)	131(52.8)	
Positive	81	37(45.7)	44(54.3)	
Tumor stage				0.589
IA, IB	63	33(52.4)	30(47.6)	
IIA, IIB	86	38(44.2)	48(55.8)	
IIIA, IIIB, IIIC	180	83(46.1)	97(53.9)	

*WD*, well-differentiated; *MD*, moderately differentiated; *PD*, poorly differentiated, *UD*, undifferentiated.

**Figure 3:**
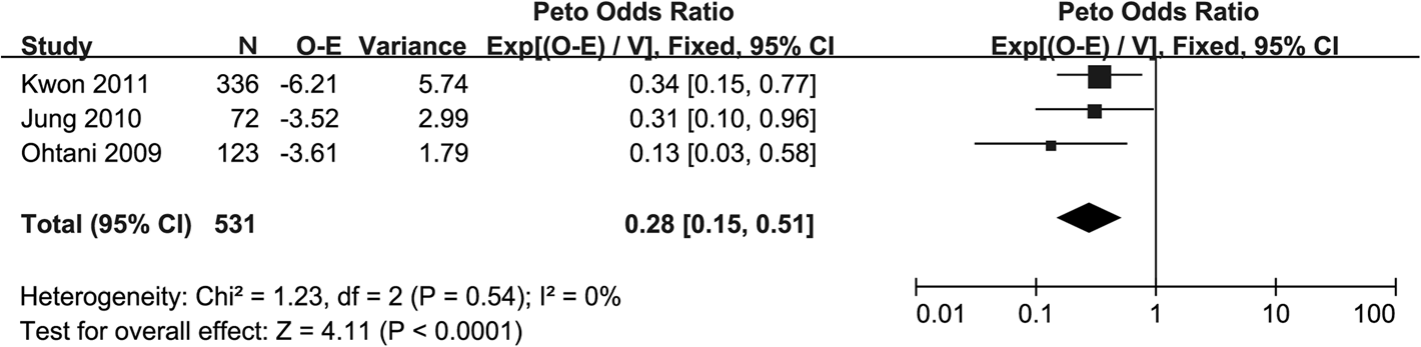
**Forest plot of the association between overall survival and the expression of claudin-4.** O-E, observed-expected.

In total 19 studies were initially identified. Fourteen studies were excluded because they did not include a prognostic comparison between high and low expression of claudin-4. One further study was excluded because critical information was missing [14]. Only one study performed the prognostic comparison based on cause-specific survival (CSS) [13], so it was excluded and we made overall survival (OS) the primary end point of our meta-analysis. Three studies met the inclusion criteria and were included in the final analysis [15,19,34]. Pooled analysis indicated that the OS of patients with high claudin-4 was better than in those with the low claudin-4 (Figure 3).

**Figure 4:**
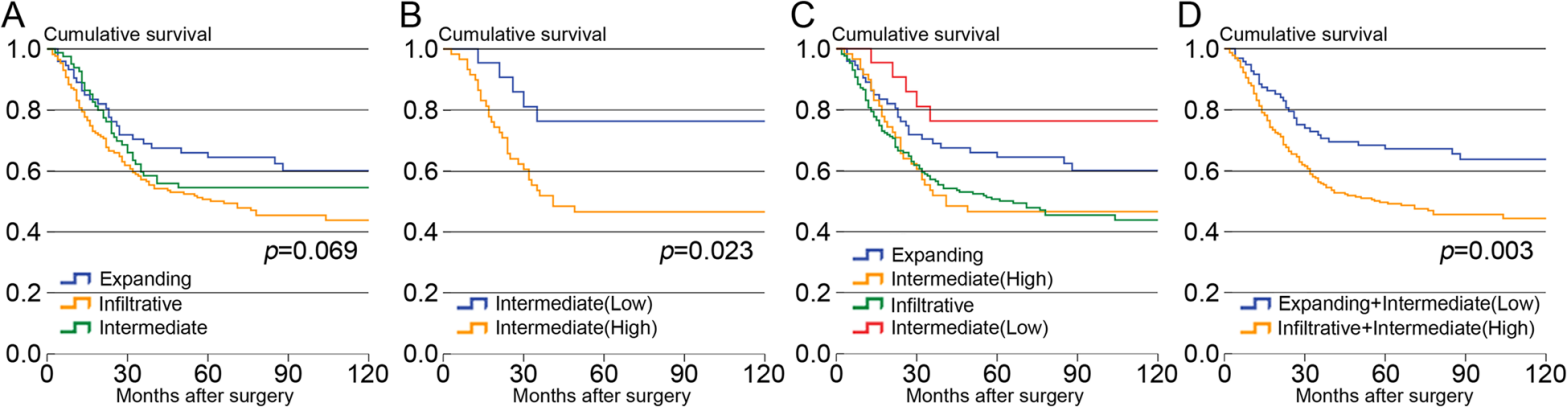
**Kaplan-Meier survival curves. (A)** Comparison of survival for three types of tumor growth pattern; **(B)** comparison of survival in patients with low and high expression levels of claudin-4 in intermediate-type growth pattern gastric cancer; **(C)** Kaplan-Meier survival curves for expanding-type, low expression levels of claudin-4 in intermediate-type, high expression levels of claudin-4 in intermediate-type, and infiltrative-type gastric cancers. **(D)** Comparison of survival in two novel subgroups.

According to univariate survival analysis, claudin protein expression was not associated with patients’ CSS (*P *= 0.637) or OS (*P *= 0.469). Stratified subclass survival analyses were performed for T and N status, lymphatic invasion, and tumor stage, and no prognostic difference was found (Additional file 1: Figure S1). In the comparison of CSS, only TNM stage (*P *< 0.001) and lymphatic invasion (*P *< 0.001) were significant prognostic factors. After Cox multivariate analysis, only TNM stage was a significant prognostic factor (*P *< 0.001). No survival difference was observed between the three types of tumor growth pattern (*P *= 0.069, Figure 4A). Further stratified analysis demonstrated no significant prognostic difference between patients who exhibited high versus low expression of claudin-4 in expanding and infiltrative type gastric cancer. However, in intermediate type growth pattern gastric cancer, the prognosis for patients exhibiting low expression levels of claudin-4 was significantly better compared to those with high expression levels (*P *= 0.023, Figure 4B). This result was also confirmed by the Cox multivariate analysis (*P* = 0.037). The five-year cancer-specific survival rate for patients with low claudin-4 expression levels in intermediate-type gastric cancer was 76.4%, which was similar to all expanding-type gastric cancers (64.5%). Our findings indicated that the five-year CSS rate for patients exhibiting high expression levels of claudin-4 in intermediate-type gastric cancer was 46.6%, which was similar to infiltrative-type gastric cancers (50.7%) (Figure 4C). Through the staining of claudin-4 in the intermediate type, we reclassified the low expression of claudin-4 into expanding type and high expression of claudin-4 into infiltrative type and composed two novel subgroups. There was a significant difference in prognosis between these two novel subgroups(*P* = 0.003, Figure 4D). After subclass survival analysis stratified by T status, N status, lymphatic invasion and tumor stage, we found that the prognostic differences of two novel subgroups were significant in the pT3/4, LN(+), stage III, lymph invasion(−) (Additional file 2: Figure S2). In multivariate analysis, the novel classification was a significant prognostic factor (*P* = 0.007).

## Discussion

The claudin family of proteins plays an important role in the maintenance of TJ function, and the expression levels often exhibit a tissue-specific pattern. Recently, an accumulating number of studies have demonstrated ectopic or aberrant expression of claudins in many tumor types [25,32,35-37]. Among the claudin subtypes, the expression of claudin-4 is frequently altered in various tumor tissues. Claudin-4 is an integral membrane protein that belongs to the claudin family. This protein is a component of TJs, and is critical for sealing cellular sheets and controlling paracellular ion flux [10].

Relatively few studies have examined the expression levels of claudin-4 in precursor lesions. Cunningham *et al*. [38] reported a 15% expression level of claudin-4 in the normal stomach, whereas in both intestinal metaplasia and dysplasia the expression of claudin-4 reached 100%. Matsuda *et al*. [36] also reported that claudin-4 was detected in the epithelium of intestinal metaplasia but not in normal epithelium. Our staining results of precursor lesion samples were in concordance with these studies. The high expression rate of claudin-4 in normal gastric mucosa was very low (only 15.9%), whereas intestinal metaplasia lesions and gastric epithelial dysplasia lesions exhibited a high expression level close to 100%. Because *CLDN-4* is expressed at high levels in the normal small intestine and colon [11], its increased expression in intestinal metaplasia is easily comprehended. However, the differential expression of claudin-4 in normal mucosa and cells exhibiting dysplasia remains unclear. The primary morphological features of epithelial dysplasia are cellular atypia, abnormal differentiation, and disorganized mucosal architecture; these changes are potentially associated with elevated claudin-4 expression. The specific underlying mechanisms need to be further elucidated. Taken together, our findings indicate that claudin-4 could potentially serve as a molecular marker of intestinal metaplasia and dysplasia in gastric mucosa.

In the present study, we found that decreased expression of claudin-4 was significantly associated with histological differentiation in gastric cancer. The differentiated group exhibited a higher expression rate of claudin-4 compared to the undifferentiated group. Lee *et al*. [18] reported that reduced expression of claudin-4 correlated with disruption of glandular structure and loss of differentiation, which was in concordance with our results.

The role of claudin-4 for prognosis remains controversial. Resnick*et al*. [13] reported that increased claudin-4 expression was a poor prognostic factor for CSS in 146 patients, and Soini *et al*. [14] proposed that claudin-4 did not associate with OS. However, the results of the meta-analysis of this study suggest that OS of patients with high claudin-4 was better than that of patients with low claudin-4. The results survival analysis based on patients in our institution indicated that claudin-4 was not associated with CSS or OS, which was similar to the results of Soini *et al*., but different from other studies.

Some previous studies have suggested that upregulation of certain claudins potentially contributes to neoplasia by directly altering TJ function [39]. Overexpression of claudin-3 and 4 may lead to an increase in invasion, motility and tumor cell survival [25]. On the contrary, in pancreatic carcinoma, overexpression of claudin-4 has been associated with significantly reduced invasiveness both *in vitro* and *in vivo*[40]. Although our study comprises nearly the largest number of cases at present, the prognostic role of claudin-4 in gastric cancer remains ambiguous (*P *= 0.637). It is possible that different populations and different environments contribute to these different results. Further study including a greater number of samples is warranted.

**Figure 5:**
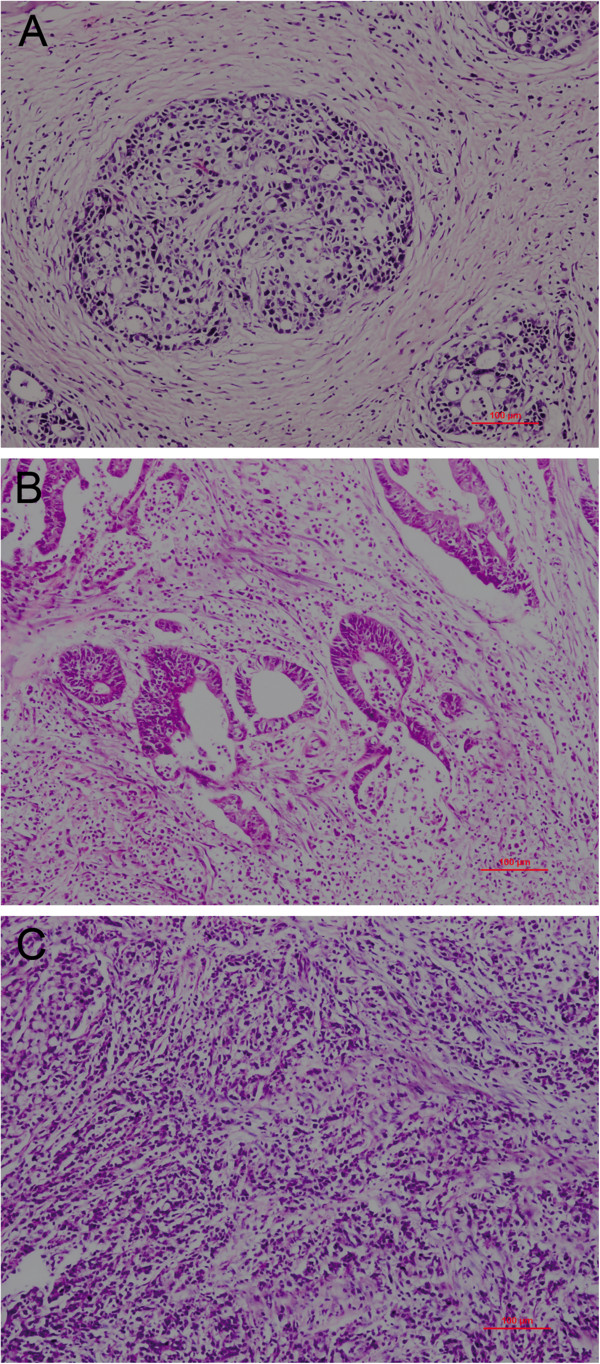
**Hematoxylin-eosin staining.** The three different growth patterns of gastric cancer: **(A)** expanding type; **(B)** intermediate type; **(C)** infiltrative type. Magnification × 100.

Histological growth patterns are important parameters for assessment of the biological behavior of gastric cancer. Based on patterns of growth and invasiveness, Ming [41] reported two types of gastric cancer, the expanding and the infiltrative type.The Japanese Gastric Cancer Association divided gastric cancer into three types based on tumor-infiltrating (INF) growth into surrounding tissue: in INFa, the tumor displays expanding growth with a distinct border from the surrounding tissue; in INFb, the tumor shows an intermediate pattern between INFa and INFc; in INFc, the tumor displays infiltrative growth with no distinct border with the surrounding tissue [42]. This final type is closer to our clinical practice, in which the intermediate type exists and is difficult to classify (Figure 5). In our results, there were differences in survival between the three types, but not significant (*P *= 0.069). Survival rates in patients with the intermediate type were between the expanding and the infiltrative types. Interestingly, in stratified analysis we found that high claudin-4 expression was associated with poor prognosis in the intermediate growth pattern (*P *= 0.023). The five-year survival rate with low expression of claudin-4 in the intermediate type (76.4%) was similar to the expanding type (64.5%), while the group with high expression of claudin-4(46.6%) was closer to the infiltrative type (50.7%). Thus, we could potentially classify intermediate-type patients according to the staining of claudin-4. We reclassified patients with low expression of claudin-4 in the intermediate group as having the expanding type, and patients with high expression of claudin-4 as having the infiltrative type of tumor. Both the univariate and multivariate analyses confirmed that there were significant differences in survival between these two subgroups. After stratified subclasses survival analysis, we found that the prognostic differences in the two novel subgroups were significant in the pT3/4, LN(+), stage III, lymph invasion(−). Although no significant difference was found in other subclasses, a certain trend still existed and we considered that the negative results of the statistical analysis were because of the relatively small sample size. Thus, the expression of claudin-4 could potentially be utilized as a basis to further identify gastric cancers of the intermediate type.

The biological role of claudin-4 in gastric cancer remains unclear. It has been previously reported that *CLDN18* is specifically expressed in normal gastric mucosa, but not *CLDN4*[11,22]. Claudin-4 is upregulated in gastric adenocarcinomas, and increased claudin-4 expression is more commonly seen in intestinal-type as opposed to diffuse-type tumors [13,17]. However, claudin-18 is downregulated in intestinal-type gastric cancer [22]. These results suggest that the distribution and expression levels of claudin proteins may vary in different cells and tissues of the body [43]. Ectopic expression of claudin is potentially associated with tumor progression. Further studies are warranted to elucidate the function of claudin-4 in the progression of gastric cancer.

## Conclusions

We demonstrated upregulation of claudin-4 in intestinal metaplasia and gastric epithelial dysplasia, which suggests its potential utility as a biomarker in gastric adenocarcinoma precursor lesions. Expression of claudin-4 was not associated with survival, but it was associated with poor histological differentiation and infiltrative patterns of tumor growth. Moreover, this study demonstrated that expression of claudin-4 could potentially be utilized as a basis to further identify gastric cancers of the intermediate type.

## Abbreviations

AEC: 3 amino-9-ethyl carbazole; AJCC: American Joint Committee on Cancer; CLDN: Claudin gene; CSS: cause-specific survival; FFPE: formalin-fixed and paraffin-embedded; HR: hazard ratio; INF: tumor infiltrating; IS: immunoreactivity score; MeSH: medical subject headings; MMP: matrix metalloproteinase; OS: overall survival; TJ: tight junction; TNM: tumor-node-metastasis.

## Competing interests

The authors declare that they have no competing interests.

## Authors’ contributions

ZNW, YY X and HMX designed the study, JLZ and ALL carried out the Immunohistochemistry, and JLZ, PG, YXS and ZNW drafted the manuscript. PG and MXW participated in the design of the study and performed the statistical analysis. All authors read and approved the final manuscript.

## Supplementary Material

Additional file 1: Figure S1 Subclass survival analysis stratified by T status, N status, lymphatic invasion and tumor stage according to the staining of claudin-4. Comparison of the survival between patients with low expression levels of claudin-4 and high expression levels in pT1-pT2 (A), pT3-pT4 (B), LN(−) (C), LN(+) (D), stage I (E), stage II (F), stage III (G), lymphatic invasion(−) (H), and lymphatic invasion(+) (I). Click here for file

Additional file 2: Figure S2 Subclass survival analysis stratified by T status, N status, lymphatic invasion and tumor stage according to the two novel subgroups. A-B. Comparison of the survival between infiltrative+intermediate(H)vs. expanding+intermediate(L) in pT1- pT2 (A), pT3-pT4 (B), LN(−) (C), LN(+) (D), stage I (E), stage II (F), stage III (G), lymphatic invasion(−) (H), and lymphatic invasion(+) (I). Click here for file
