# Supplementary figures and images for: Clinicopathological significance of claudin-4 in gastric carcinoma
Source: World J Surg Oncol. 2013 Jul 4;11:150. doi: 10.1186/1477-7819-11-150 (PMC3717126; doi:10.1186/1477-7819-11-150)

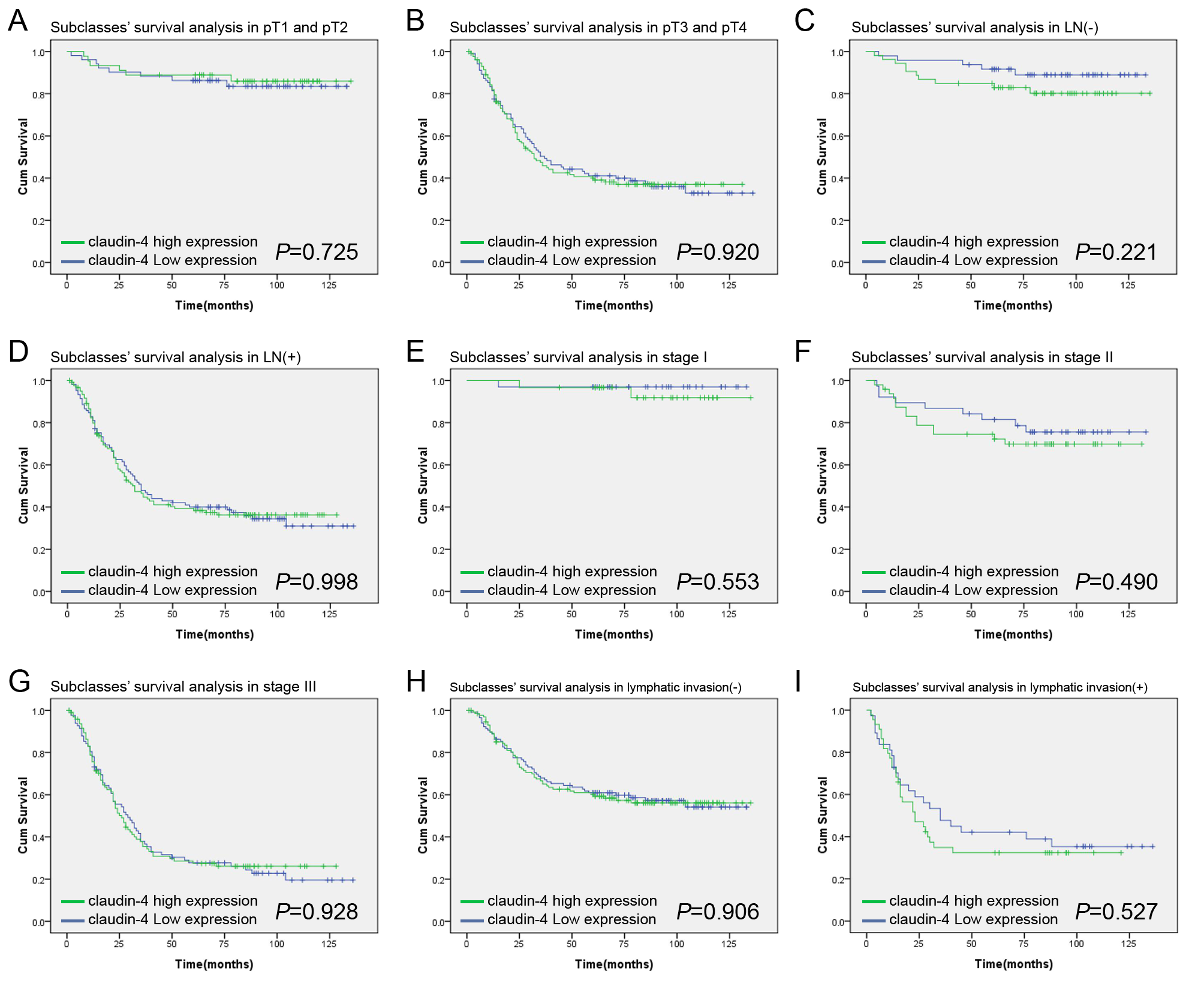

Supplement: Additional file 1: Figure S1 — Subclass survival analysis stratified by T status, N status, lymphatic invasion and tumor stage according to the staining of claudin-4. Comparison of the survival between patients with low expression levels of claudin-4 and high expression levels in pT1-pT2 (A), pT3-pT4 (B), LN(−) (C), LN(+) (D), stage I (E), stage II (F), stage III (G), lymphatic invasion(−) (H), and lymphatic invasion(+) (I). [file 1477-7819-11-150-S1.tiff]

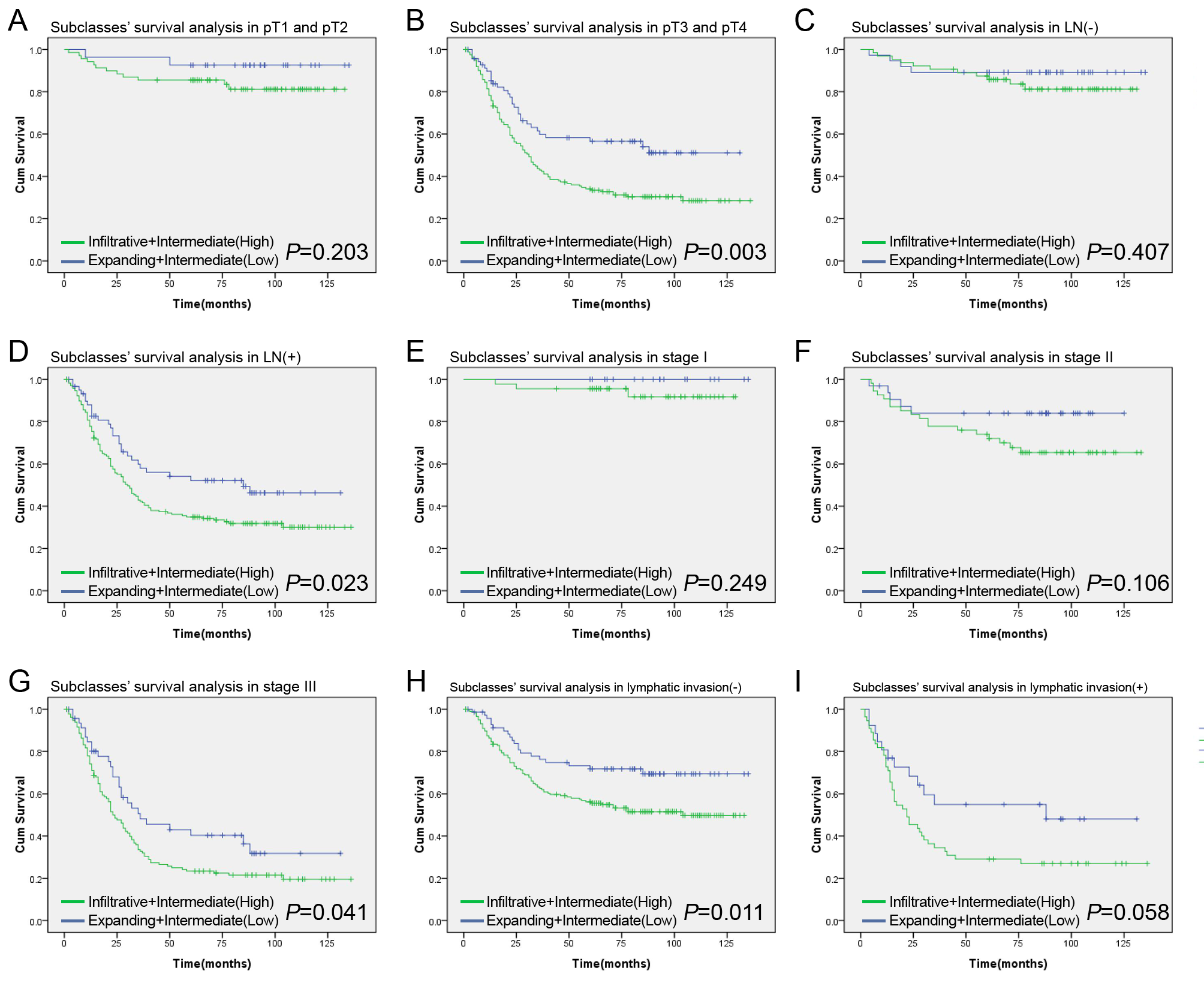

Supplement: Additional file 2: Figure S2 — Subclass survival analysis stratified by T status, N status, lymphatic invasion and tumor stage according to the two novel subgroups. A-B. Comparison of the survival between infiltrative+intermediate(H)vs. expanding+intermediate(L) in pT1- pT2 (A), pT3-pT4 (B), LN(−) (C), LN(+) (D), stage I (E), stage II (F), stage III (G), lymphatic invasion(−) (H), and lymphatic invasion(+) (I). [file 1477-7819-11-150-S2.tiff]
